# The Polar Fluid Model for Blood Flow through a Tapered Artery with Overlapping Stenosis: Effects of Catheter and Velocity Slip

**DOI:** 10.1155/2015/174387

**Published:** 2015-02-28

**Authors:** J. V. Ramana Reddy, D. Srikanth

**Affiliations:** Department of Applied Mathematics, Defence Institute of Advanced Technology (Deemed University), Pune 411025, India

## Abstract

The blood flow through an overlapping clogged tapered artery in the presence of catheter is discussed. Since cholesterol deposition is resulting in the stenosis formation, velocity slip at the arterial wall is considered. The equations governing the fluid flow have been solved analytically under the assumption of the mild stenosis. The analysis with respect to various parameters arising out of fluid and geometry considered, on physiological parameters such as impedance and wall shear stress at the maximum height of the stenosis as well as across the entire length of the stenosis has been reported. A table summarizing the locations of extreme heights and the corresponding annular radii is provided. It is observed that the wall shear stress is the same at both the locations corresponding to the maximum height of the stenosis in case of nontapered artery while it varies in case of tapered artery. It is also observed that slip velocity and diverging tapered artery facilitate the fluid flow. Shear stress at the wall is increasing as micropolar parameter is decreasing and the trend is reversed in case of coupling number. The results obtained are validated by comparing them with the experimental and theoretical results.

## 1. Introduction

The cardiovascular system permits blood and bodily fluid to transport nutrients, gas, and hormones to any or all elements of the body and collects the waste product, deoxygenated blood from all elements of the body, thus enabling all organs of the body to work efficiently. In this world majority of the deaths are associated with vessel diseases. Among them maximum deaths are related to the characteristics of blood flow and vessel moments. In particular the circulatory connected issues are the major causes of health problems and deaths in the present world. This is supported by the report of the World Health Organization (WHO), according to which, seventeen million deaths in 2008 are associated with the heart. The basic cause of heart related disorders is due to occlusion, which compromise the functioning of the vital organs. Most notably, the heart and brain are often affected, as happens in MI (destruction of heart tissue resulting from obstruction of the blood supply to the heart muscle) and stroke.

The deposition of excessive fatty components and abnormal intravascular growth within the lumen of the artery results in the formation of the stenosis. This results in the narrowing of the artery and this condition is known as atherosclerosis. Formation of the stenosis results in the obstruction of the flow of blood thus resulting in abnormal variations of the blood flow characteristics. Catheterization is a method in which a long skinny versatile tube is inserted into the stenotic artery to treat atherosclerosis. Insertion of such a tube (called catheter) into the lumen of the artery results in significant changes in the blood flow, even in massive arteries. Therefore, the study of catheter effects in physiological fluid flow through a stenotic artery is vital. Hence the mathematical understanding of such a scenario was taken up by many assorted researchers. In particular understanding of biological development of blood flow through stenosed arteries has become prominent and has played a leading role in the diagnosis and the treatment of heart related diseases.

Huo and Fu [1] reported about the advances that have taken place in clinical technologies, in the field of respiratory mechanics, exchange of gas in lungs, and so forth. Lykoudis and Roos [2] studied the mechanics of the duct with an inserted catheter by considering a moving peristaltic wave on the stationary cylinder. Smith [3] studied the flow through constricted tube and analyzed the impact of mild, moderate, and severe stenoses on totally developed flow. He developed a mathematical model for understanding the dynamics of blood flow in an exceedingly constricted artery. Back [4] obtained the influence of catheter radius on resistance to the flow and pressure drop. In his analysis he used an artificial set-up and conducted experiments using 33% sugar-water liquid.

All the above mentioned studies modelled blood as a classical Newtonian fluid. Because of the suspension nature of the blood cells, human blood exhibits non-Newtonian behavior at low shear rates. Dash et al. [5] discussed both steady nature and pulsatile nature of the flow by considering blood as casson fluid. He considered the presence of periodic pressure gradient and solved the resulting equations analytically by using the perturbation technique. He concluded that because of the yield stress, the insertion of the catheter in the nursing artery leads to the formation of two yield planes inside the flow region. Power law fluid flow through a circular region with porous walls was studied by Devanathan and Raju [6]. The spiral flow through a regular constricted artery has been analyzed by Linge et al. [7]. Here he showed that spiral flow has additional stability in the downstream region when compared to that of the traditional flow. Srinivasacharya and Srikanth [8] analyzed the incompressible couple stress fluid flow through the mild simple stenosis in the presence of the catheter. Here they concluded that the couple stress fluid offers more resistance to the flow than that offered by the Newtonian fluid. Ramana et al. [9, 10] discussed steady and pulsatile nature of polar fluid through asymmetric tapered catheterized artery. Here they have given a table showing the locations of the maximum height of the stenosis and the corresponding annular radius for various values of the tapered and shape prameter.

Among the several non-Newtonian fluids present, the micropolar fluid encompasses special importance as it exhibits microrotational inertia and microrotational effects which arise from the naive structure of the fluid. This micropolar fluid model can adequately represent fluids consisting of dipole elements and can support couple stress and body couple effects as well. The classical Navier-Stokes theory is incapable of predicting these new physical phenomena. Since Blood consists of dipole elements, it seems very appropriate to model blood using micropolar fluid. The angular velocity at any point in the extended Navier-Stokes equation as described by Hansen et al. [11] is given by 1/2 curl $\bar{v}$ and is independent of the microrotation vector appearing in the micropolar fluid model. Both these quantities will coincide at the boundary. A detailed literature and development of the model of micropolar fluid and their applications are given by Lukaszewicz [12] and Eringen [13]. Srinivasacharya and Srikanth [14] studied the steady flow of micropolar fluid through mild symmetric stenosis in the presence of the catheter. Mekheimer and El Kot [15] considered blood as micropolar fluid and discussed the effects of the asymmetry nature of the stenosis in their steady flow analysis.

A lot of work has been done in relation to the development of sclerosis, wherein the majority of the studies, shape of the stenosis was considered to be either symmetric or asymmetric. But it is understood that the stenosis may be multiple in nature or develop in an irregular manner. Further, stenosis may be overlapping or composite in nature. Haldar [16] investigated the impact of the various forms of the stenosis on the resistance to the flow in case of power-law fluid. Srivastava et al. [17] discussed the effects of the catheter on blood flow through an overlapping stenotic artery. Here a two-phase macroscopic model of the blood was considered. Blood flow through overlapping stenosed arteries was also considered by Riahi et al. [18]. In particular Daniel and his group investigated the hematocrit and constriction effects on impedance, shear stress at the boundary. There is no doubt that tapering in arteries is vital in the mammalian arterial system and the formation of stenosis on the tapered wall, may alter the flow dynamics to a great extent. Understanding the impact of tapered arteries on flow dynamics will be very useful in the design of prosthetic blood vessels which has surgical advantages. Mekheimer and El Kot [19, 20] and Mandal [21] considered the tapering effects in their studies.

In all the above studies usual no-slip condition at the arterial wall has been considered. However variety of studies of suspensions of blood flow, have established the likely presence of slip at the boundary. In particular Brunn [22] theoretically justified the presence of slip and Bennett [23] did the same experimentally. In fact it has been shown that even when the red cells are in contact with the wall they slip freely. Recently Misra and Shit [24] considered velocity slip at the arterial boundary to understand the effect of skin friction, impedance, and so forth by considering Herschel-Bulkley fluid model.

Hence in this paper we intend to explore the effects of overlapping stenosis on the physiological parameters of the blood flow that is modelled using micropolar fluid through a catheterized tapered artery. Velocity slip at the wall is additionally thought of. The analysis is done analytically. This configuration and the related results could be very useful in the development of various clinical related technologies and equipments. It is also useful to the scientists engaged in the design and development of artificial organs.

## 2. Formulation of the Problem

### 2.1. Schematic Representation of Overlapping Stenosis

**Figure 1:**
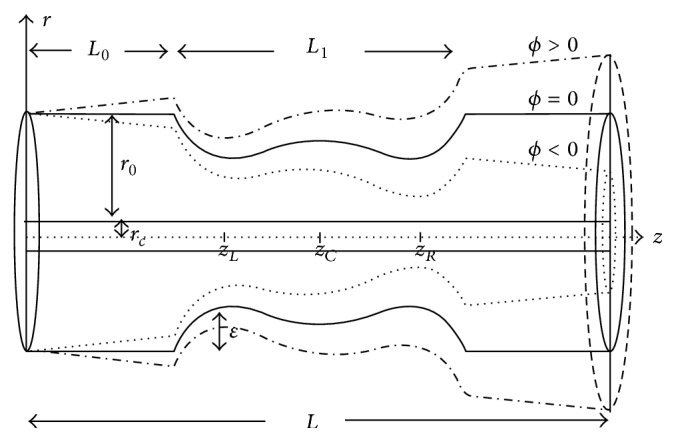
Overlapping stenotic tapered artery with catheter.

The schematic diagram of the overlapping stenotic tapered artery in the presence of catheter is as shown in Figure 1. Here blood is modelled by using incompressible micropolar fluid which is flowing in an annular region formed by two coaxial cylinders represented by an artery and a catheter. The radius of the artery is varying while radius of the catheter *r*_*c*_ is fixed. *r*_0_ is the annular radius in the nonstenotic portion for nontapered artery. *ζ*( = tan(*ϕ*)) is the tapered parameter with *ϕ* being the tapering angle. *ϕ* < 0, *ϕ* > 0, and *ϕ* = 0 correspond to converging, diverging and nontapered artery. Here *z*_*L*_ and *z*_*R*_ correspond to the locations where maximum heights of the stenosis is occurring. *z*_*C*_ indicates the location on *z*-axis corresponding to the critical height of the overlapping stenosis.

The mathematical form of the geometry of the overlapping stenotic tapered artery is as described by [17, 19], which is given by

(1) hz=r0+ζz−32ϵL14 ·11z−L0L13−47z−L02kkkk·L12+72z−L03L1kkkk−36z−L04,L0≤z≤L0+L1r0+ζzotherwise,

where *L*_1_ is the length of the stenosis. *ϵ* is the maximum height of the stenosis and the locations of the maximum heights of the stenosis are dependent on *ζ*. The locations of the extreme heights of the stenosis are obtained as the roots of the cubic equation

(2) 144z−L03−216L1z−L02+94L12z−L0 −11L13+2ζL143ϵ=0.

### 2.2. Mathematical Model

The balance equations which govern the flow of micropolar fluid are

(3) DρDt+ρ∇·V¯=0,ρDV¯Dt=−∇P−μ+κ∇×∇×V¯ +κ∇×ω¯+λ+2μ∇∇·V¯+ρf,ρjDω¯Dt=−2κω¯+κ∇×V¯−γ∇×∇×ω¯ +α+β+γ∇∇·ω¯+ρl,

where the velocity vector is $\bar{V}$, the microrotation vector is $\bar{\omega }$, *P* is the fluid pressure. *j*, *ρ* are the microinertia moment and density parameters, respectively, while *f* and *l* are body force and body moment, respectively. The material constants *α*, *β*, *γ*, *μ*, and *κ* satisfy the following inequalities:

(4) $$\begin{array}{c} 3\alpha +\beta +\gamma \geq 0,\;\gamma \geq \left|\beta \right|, \end{array}$$

(5) $$\begin{array}{c} 2\mu +\kappa \geq 0,\;\kappa \geq 0. \end{array}$$

In the absence of body force and body couple the balance equations (3) of incompressible steady micropolar fluid get reduced to

(6) ∇·V¯=0,ρV¯·∇V¯=−∇P−μ+κ∇×∇×V¯+κ∇×ω¯,ρjV¯·∇ω¯=−2κω¯+κ∇×V¯−γ∇ ×∇×ω¯+α+β+γ∇∇·ω¯.

The problem has been studied in cylindrical polar coordinate system (*r*, *θ*, *z*) with *r* = 0 as the axis of symmetry. Since the flow is assumed to be axisymmetric, all the flow variables are independent of *θ*. The velocity vector $\bar{V}$ is given by $\bar{V}=(v_{r},0,v_{z})$ and the microrotation vector is  $\bar{\omega }=(0,\nu_{\theta },0)$, where *v*_*r*_, *ν*_*θ*_, and *v*_*z*_ are functions of *r* and *z*.

Under the above assumptions, (6) will get reduced to

(7) ∂vr∂r+vrr+∂vz∂z=0,ρvr∂vr∂r+vz∂vr∂z=−∂p∂r−κ∂νθ∂z+μ+κ ·∂2vr∂z2+∂2vr∂r2+1r∂vr∂r−vrr2,ρvr∂vz∂r+vz∂vz∂z =−∂p∂z+μ+κ  ·∂2vz∂z2+∂2vz∂r2+1r∂vz∂r+κ1r∂rνθ∂r,ρjvr∂νθ∂r+vz∂νθ∂z =−2κνθ+γ∂2νθ∂z2+∂∂r1r∂rνθ∂r  +κ∂vr∂z−∂vz∂r.

The nondimensional variables are defined as

(8) $$\begin{array}{c} z^{\prime }=\frac{z}{L_{1}},\;\;v_{z}^{\prime }=\frac{v_{z}}{u_{0}},\;\;v_{r}^{\prime }=\frac{L_{1}v_{r}}{u_{0}\epsilon },\\ h^{\prime }=\frac{h}{r_{0}},\;\;p^{\prime }=\frac{r_{0}^{2}p}{u_{0}L_{1}\mu },\;\;\tau_{rz}^{\prime }=\frac{r_{0}\tau_{rz}}{u_{0}},\\ \tau_{zr}^{\prime }=\frac{r_{0}\tau_{zr}}{u_{0}},\;\;\epsilon^{\prime }=\frac{\epsilon }{r_{0}},\\ r^{\prime }=\frac{r}{r_{0}},\;\;\lambda^{\prime }=\frac{\lambda }{L},\\ j^{\prime }=\frac{j}{r_{0}^{2}},\;\;\nu_{\theta }^{\prime }=\frac{r_{0}\nu_{\theta }}{u_{0}},\;\;\zeta^{\prime }=\frac{\zeta L_{1}}{r_{0}}, \end{array}$$

where *u*_0_ is the typical axial velocity.

Introducing the above nondimensional variables into (7) and dropping dashes, we get

(9) ξ∂vr∂r+vrr+∂vz∂z=0,Reδ2ξδvr∂vr∂r+vz∂vr∂z =−∂p∂r−δ2N1−N∂νθ∂z+ξδ21−N  ·δ2∂2vr∂z2+∂2vr∂r2+1r∂vr∂r−vrr2,Reδξvr∂vz∂r+vz∂vz∂z =−∂p∂z+N1−N1r∂rνθ∂r  +11−Nδ2∂2vz∂z2+1r∂∂rr∂vz∂r,jRe1−NNδξvr∂νθ∂r+vz∂νθ∂z =−2νθ+δ2ξ∂vr∂z−∂vz∂r  +2−Nm2δ2∂2νθ∂z2+∂∂r1r∂rνθ∂r,

where *N* = *κ*/(*μ* + *κ*) is the coupling number (0 ≤ *N* < 1), *m* ^2^ = [*r*_0_^2^(2*μ* + *κ*)*κ*]/[*γ*(*μ* + *κ*)] is the micropolar parameter, *Re* = (*ρu*_0_ *r*_0_)/*μ* is the Reynolds number, and *δ* = *r*_0_/*L*_1_ and *ξ* = *ϵ*/*r*_0_.

Under the assumptions of mild stenosis; that is, *ξ*( = *ϵ*/*r*_0_) ≪ 1 and subject to the additional condition *δ*( = *r*_0_/*L*_1_) ≈ *O*(1), we find that ∂*p*/∂*r* ≪ ∂*p*/∂*z* and ∂*ν*_*θ*_/∂*z* ≪ ∂*ν*_*θ*_/∂*r*.

Hence (9) get reduced to

(10) $$\begin{array}{c} \frac{\partial v_{z}}{\partial z}=0, \end{array}$$

(11) $$\begin{array}{c} \frac{\partial p}{\partial r}=0, \end{array}$$

(12) $$\begin{array}{c} \frac{\partial p}{\partial z}=\frac{N}{1-N}\left(\frac{1}{r}\frac{\partial \left(r\nu_{\theta }\right)}{\partial r}\right)+\frac{1}{1-N}\left[\frac{1}{r}\frac{\partial }{\partial r}\left(r\frac{\partial v_{z}}{\partial r}\right)\right], \end{array}$$

(13) $$\begin{array}{c} 2\nu_{\theta }=-\frac{\partial v_{z}}{\partial r}+\frac{2-N}{m^{2}}\left[\frac{\partial }{\partial r}\left(\frac{1}{r}\frac{\partial \left(r\nu_{\theta }\right)}{\partial r}\right)\right]. \end{array}$$

The dimensionless form of (1) is

(14) h(z)=1+ζz−3ϵ2 ·11z−γ−47z−γ2+72kiik·z−γ3−36z−γ4,if L0<z<L0+L1,1+ζz,otherwise,

where, *γ* = *L*_0_/*L*_1_.

The nondimensional form of (2), from which the various locations of *z*_*L*_, *z*_*C*_, and *z*_*R*_ for various values of tapered parameter are calculated, is given by

(15) 144z−γ3−216z−γ2+94z−γ −11+2ζL13ϵ=0.

The locations of the extreme heights of the stenosis (*z*_*L*_, *z*_*C*_, and *z*_*R*_) and the corresponding radii of the annular region for various values of the tapered parameter *ζ* are numerically calculated with fixed values of *L* = 2,  *L*_1_ = *L*/2,  *L*_0_ = (*L* − *L*_1_)/2,  *r*_0_ = 1. The values are summarized in the form of Table 1.

The dimensionless boundary conditions are given by

(16) $$\begin{array}{c} v_{z}=u,\;\nu_{\theta }=0\;\text{at}\;\;r=h\left(z\right),\\ v_{z}=0,\;\nu_{\theta }=0\;\text{at}\;\;r=r_{c}, \end{array}$$

where *u* is the velocity slip at the wall of the artery.

## 3. Solution of the Problem

From (11), it is observed that the pressure is invariant in the radial direction. Further (12) can be rewritten as

(17) $$\begin{array}{c} \frac{\partial v_{z}}{\partial r}=\left(1-N\right)\left(\frac{c_{1}}{r}+\frac{r}{2}\frac{\partial p}{\partial z}\right)-N\nu_{\theta }. \end{array}$$

Substituting (17) in (13), we get

(18) $$\begin{array}{c} \frac{\partial^{2}\nu_{\theta }}{\partial r^{2}}+\frac{1}{r}\frac{\partial \nu_{\theta }}{\partial r}-\left(m^{2}+\frac{1}{r^{2}}\right)\nu_{\theta }=\frac{\left(1-N\right)m^{2}}{\left(2-N\right)}\left[\frac{c_{1}}{r}+\frac{r}{2}\frac{\partial p}{\partial z}\right]. \end{array}$$

The solution of the above equation is

(19) $$\begin{array}{c} \nu_{\theta }=c_{2}I_{1}\left(mr\right)+c_{3}K_{1}\left(mr\right)-\frac{\left(1-N\right)}{\left(2-N\right)}\left[\frac{c_{1}}{r}+\frac{r}{2}\frac{\partial p}{\partial z}\right]. \end{array}$$

Using (19) in (17), we get

(20) vz=Nm−c2I0mr+c3K0mr −21−N2−Nc1ln⁡⁡r+r24∂p∂z+c4,

where *I*_1_(*mr*), *K*_1_(*mr*), *I*_0_(*mr*), and *K*_0_(*mr*) are the modified Bessels functions of the first order and zeroth order of first and second kind, respectively. *c*_*i*_, *i* = 1,2, 3,4 are the constants which are evaluated by using the boundary conditions (16).

The nondimensional volumetric flow is obtained using *Q* = ∫_*r*_*c*__^*h*(*z*)^2*rv*_*z*_ *dr* in the following simplified form

(21) $$\begin{array}{c} Q=2\frac{\partial p}{\partial z}F\left[h\left(z\right)\right], \end{array}$$

where

(22) Fhz=−Nm2d2hzI1(mhz)−rcI1mrc kkkkkk+d3hzK1mhz−rcK1mrc +1−N2−R ·∂p∂zhz4−rc48  k+d1hz2ln⁡hz−12kkkkkkkkkkhz4−rc48−rc2ln⁡rc−12 +d4hz2−rc22,

with *d*_*i*_ = *c*_*i*_/(∂*p*/∂*z*) and *i* = 1,2, 3 and 4.

The pressure difference Δ*p* across the artery is obtained from (21) using

(23) $$\begin{array}{c} \Delta p=\frac{Q}{2}\overset{L}{\underset{0}{\int }}\frac{1}{F\left[h\left(z\right)\right]}dz. \end{array}$$

### 3.1. The Resistance to the Flow (Impedance)

The impedance is directly related to the flow rate of the fluid and has direct impact on physiological dynamics of the flow. Hence the analysis of the resistance to the flow with respect to various fluid and geometry parameters is important. The resistance to the fluid is calculated as

(24) λ=ΔPQ=12∫0L1Fhz,rcdz=12∫0L01Fhz,rcdz+∫L0L0+L11Fhz,rcdzkkkkk+∫L0+L1L1Fhz,rcdz.

The nondimensional form of the impedance is

(25) λ=Γ2∫0γ1Fhz,rcdz+∫γγ+11Fhz,rcdzkkkkk+∫γ+11/Γ1Fhz,rcdz,

where Γ = *L*_1_/*L*.

### 3.2. Wall Shear Stress

One of the basic objectives of the problems related to physiological fluid dynamics is to predict shear stress at the wall in clogged arteries. This significantly influences the rate of mass transport across the artery walls and the formation of atheroma on the walls of the arteries as in atherosclerosis. The expression for shear stress is obtained from

(26) $$\begin{array}{c} \tau_{rz}=-\frac{N}{\left(1-N\right)}\nu_{\theta }-\frac{1}{\left(1-N\right)}\frac{\partial v_{z}}{\partial r},\\ \tau_{zr}=-\frac{N}{1-N}\nu_{\theta }+\frac{\partial v_{z}}{\partial r}. \end{array}$$

Shear stress at the wall for the micropolar fluid is skew-symmetric. Further using boundary conditions (16), the nondimensional shear stress at the wall (*τ*_*w*_) is computed as

(27) τw=−11−N∂vz∂r∂vz∂rr=hz.

Therefore

(28) τw=−11−N ·n11+NG−Hn12Fc1I1n1r−c2K1n1r −11−N ·n21+NG−Hn22Fc3I1n2r−c4K1n2r.

Wall shear stress is calculated in the stenosis region and at the maximum heights of the stenosis.

## 4. Results and Discussion

The objective of this analysis is to study the change in flow pattern and estimate the variation in flow resistance and wall shear stress in a narrow overlapping stenosed tapered artery when a catheter is inserted into it. In addition to the above, velocity slip at the boundary of the artery has been considered. Blood is modelled as steady incompressible micropolar fluid. The impact on the physiological parameters is obtained for different parameters arising out of the geometry and fluid considered, such as *m* (micropolar fluid parameter), *N* (coupling number), *r*_*c*_ (radius of the catheter), *ϵ* (maximum height of the stenosis), and Γ, *ϕ* (tapering angle), and *u* (slip velocity). The results obtained here have been validated and the trend is found to be agreeing very well with the experimental and theoretical results reported in the literature.

**Figure 2:**
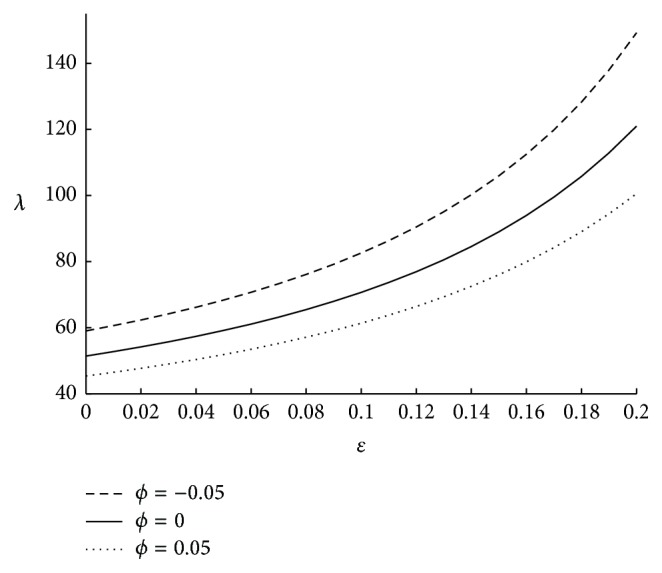
The variation of impedance with respect to *ϕ* when *N* = 0.75, *m* = 50, Γ = 0.5, *r*_*c*_ = 0.1, and *u* = 0.01.

**Figure 3:**
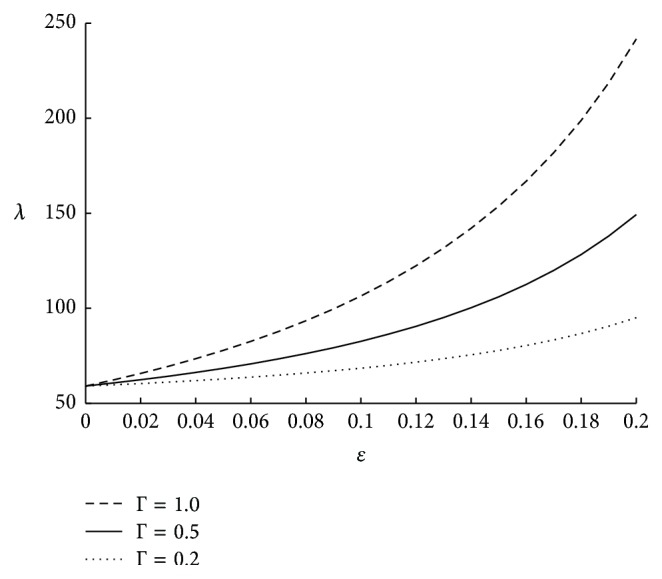
The variation of impedance with respect to Γ when *r*_*c*_ = 0.1, *N* = 0.75, *m* = 50, *u* = 0.01, and *ϕ* = −0.05.

**Figure 4:**
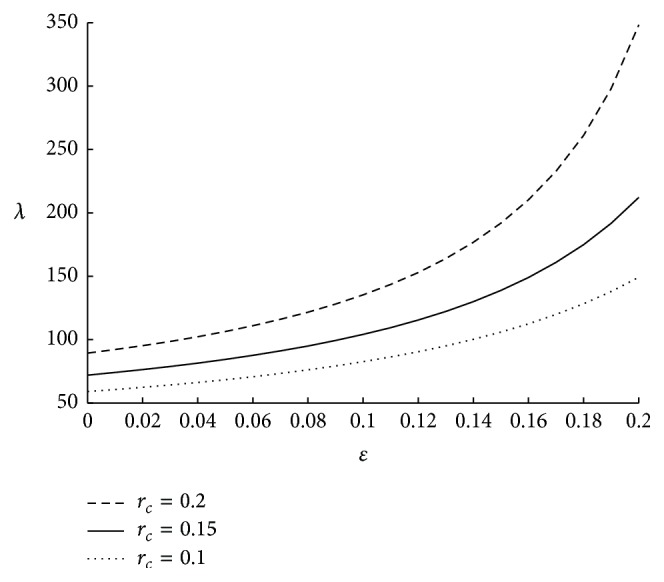
The variation of impedance with respect to *r*_*c*_ when *N* = 0.75, *m* = 50, Γ = 0.5, *u* = 0.01, and *ϕ* = −0.05.

The frictional resistance (*λ*) per unit length of the artery is calculated using (25). It is clear that under a given pressure gradient a greater resistance implies less flow of fluid. Thus the resistance gives the measure of the volume of the fluid transported in the artery.  Figure 2 underlines the importance of tapering angle *ϕ* on the resistance to the flow. Here it is observed that impedance is increasing as *ϕ* is decreasing. Hence it can be concluded that impedance is maximum in case of converging tapered artery and goes on decreasing as the tapered artery diverges. The results obtained above are in expected lines as the flow rate is less in converging artery when compared to that of diverging artery. Having understood the effect of the tapering angle all the further results are obtained in case of converging tapered artery. From Figure 3 it is observed that the length of the stenosis also significantly influences the impedance. As the length of the stenosis is increasing; that is, as the spread of the stenosis on the inner wall of the artery is increasing, the annular region is getting narrowed which leads to high variation in pressure difference, which further results in high impedance. The effect of catheter radius on impedance is shown in Figure 4. Here it is seen that impedance is high for higher values of the catheter radius. This is because as catheter radius increases the annular region get narrowed which results in high impedance. It is also observed that for few initial values of *ϵ* the increase in impedance is less and it increases exponentially for higher values of *ϵ*. Further the above results have been validated with the experimental of Back [4] and with the theoretical results for symmetric stenosis, as considered by Srinivasacharya and Srikanth [14]. Here, the results obtained by us are in similar lines with that of the experimental results, thus validating our approach. Further the present results which are obtained in case of overlapping stenosis are compared with the results obtained for symmetric stenosis. Here it is found that impedance is more in case of overlapping stenosis when compared to that of symmetric stenosis. Also impedance increases rapidly for the overlapping stenosis as the ratio of catheter radius to the radius of the annular region increases. This highlights the importance of considering overlapping stenosis which is more natural to occur. The comparision of the results is shown in the form of Table 2.

**Figure 5:**
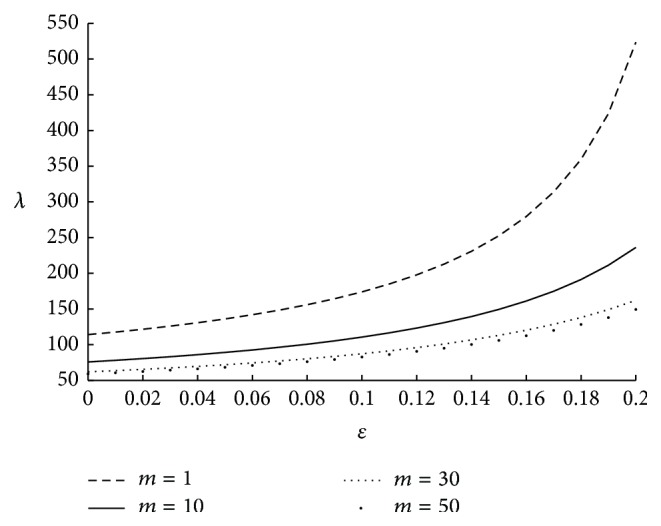
The variation of impedance with respect to *m* when *r*_*c*_ = 0.1, *N* = 0.75, Γ = 0.5, *u* = 0.01, and *ϕ* = −0.05.

**Figure 6:**
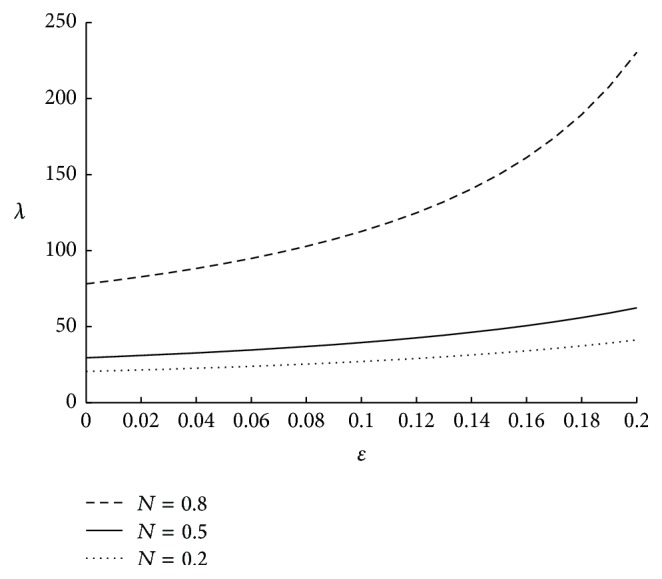
The variation of impedance with respect to *N* when *r*_*c*_ = 0.1, *m* = 50, Γ = 0.5, *u* = 0.01, and *ϕ* = −0.05.

**Figure 7:**
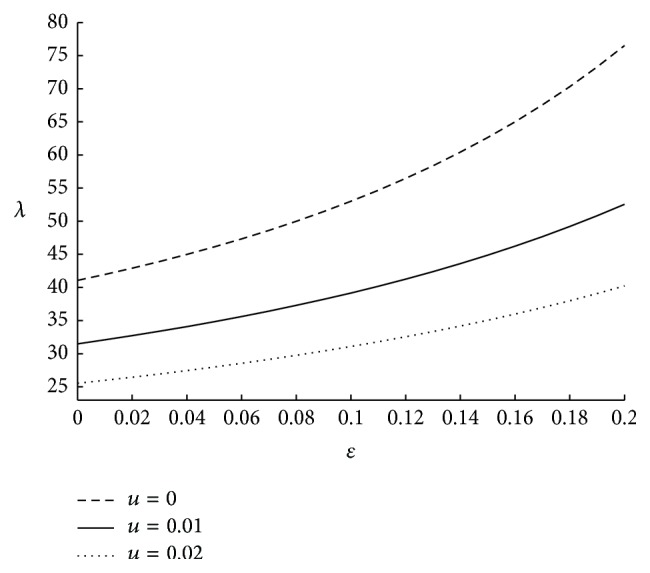
The variation of impedance with respect to *u* when *N* = 0.75, *m* = 50, Γ = 0.5, *r*_*c*_ = 0.1, and *ϕ* = −0.05.

It is to be noted that the motion of micropolar fluid may get affected by the viscous action (*μ*) of the fluid elements, couple stresses (*γ*) and the direct coupling of the microstructure to the velocity field (*κ*). All these fluid parameters can have any value greater than or equal to zero. Hence, the ratio of the couple stress to viscous effects, that is, *γ*/*μ* and microstrure coupling to the viscous effects, that is, *κ*/*μ*, can have any value greater than or equal to zero. This knowledge will help us to understand the physics behind the variation in the physiological parameters with respect to the associated fluid parameters. The influence on impedance for various values of micropolar parameter is shown in Figure 5. Here resistance to the flow is decreasing as *m* is increasing. This is because, as *m* increases the coupling effects of the fluid decrease, thus resulting in less impedance. The effect of coupling number is depicted in Figure 6, from which it is observed that impedance is increasing while coupling number is increasing. Here *N* → 0 when the ratio of microstructure coupling to the viscous effect *κ*/*μ* → 0. Hence it can be technically understood that as the microstructure coupling effects corresponding to a fluid are increasing impedance is increasing. The effect of velocity slip on resistance to the flow is sketched in Figure 7. The resistance to the fluid flow is higher in the no-slip case and it is observed that even a small slip in the velocity at the boundary, facilitates the fluid flow to a large extent thus reducing the resistance to the flow. Two different values of the velocity slip *u* = 0.01 and 0.02 are considered at the arterial wall. They correspond to 1% and 2% of the average velocity of the blood, respectively. Hence it can be concluded that even a small slip in the velocity has significant effect on resistance to the flow.

Shear stress at the wall is computed at two locations corresponding to the maximum height of the stenosis denoted by *z*_*L*_ and *z*_*R*_. Further it is very interesting to note that the location of *z*_*L*_ is dependent on the tapered parameter *ζ*. Same is the case with the locations of *z*_*R*_ which is different for different tapered parameters. The same have been computed and have been compiled in the form of Table 1. This change in the locations which is leading to change in the annular radii was not considered by any of the earlier researchers to the best of the knowledge of the present authors. Further this important hemodynamic index is calculated across the entire length of the stenosis and it's variation is also studied.

**Figure 8:**
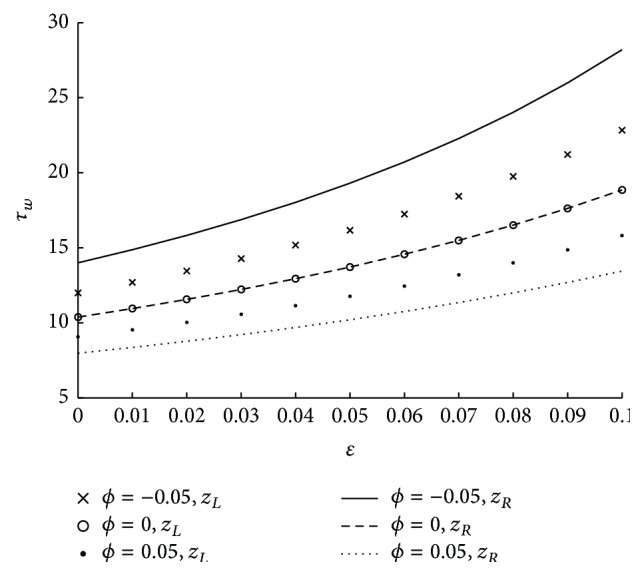
The variation of shear stress at wall with respect to *ϕ* at both maximum heights in the stenosis with fixed values of *N* = 0.75, *r*_*c*_ = 0.1, *u* = 0.01, and *m* = 50.

**Figure 9:**
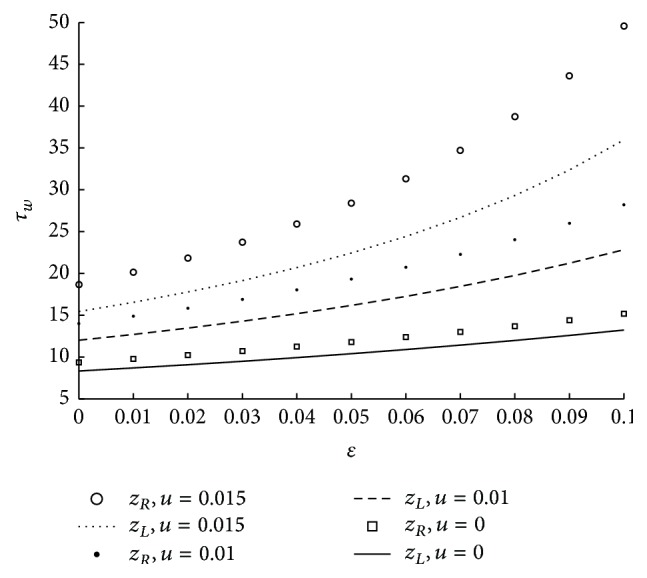
The variation of shear stress at wall with respect to *u* at both maximum heights in the stenosis with fixed values of *N* = 0.75, *r*_*c*_ = 0.1, *ϕ* = −0.05, and *m* = 50.

**Figure 10:**
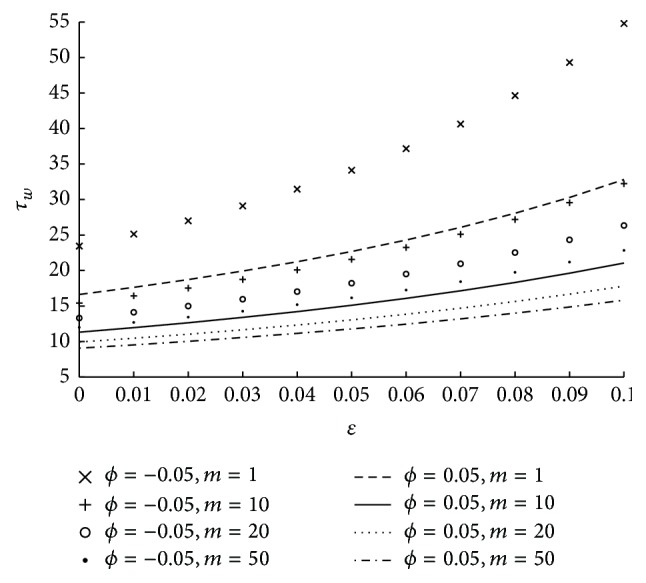
The variation of shear stress at wall with respect to *ϕ*, *m* at *z*_*L*_ when *N* = 0.75, *r*_*c*_ = 0.1, and *u* = 0.01.

**Figure 11:**
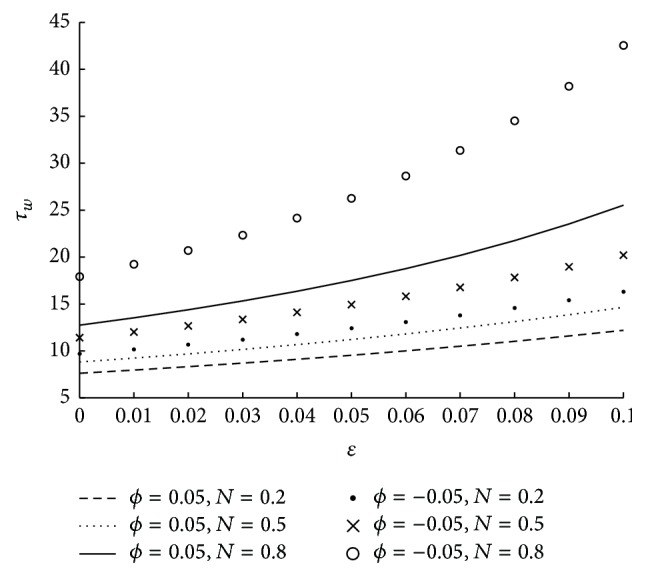
The variation of shear stress at wall with respect to *ϕ*, *N* at *z*_*L*_ when *m* = 10, *r*_*c*_ = 0.1, and *u* = 0.01.

**Figure 12:**
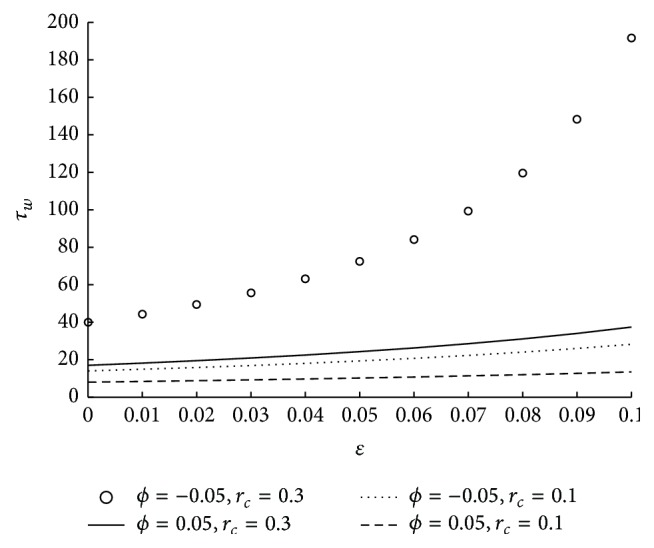
The variation of shear stress at wall with respect to *ϕ*, *r*_*c*_ at *z*_*R*_ when *m* = 50, *N* = 0.75, and *u* = 0.01.

The effect of tapered parameter at different locations of maximum height of the stenosis on wall shear stress is shown in Figure 8. Here it is observed that shear stress at the wall is more in case of converging tapered artery followed by nontapered and diverging tapered artery. Further in case of converging artery, the wall shear stress is more at *z*_*R*_ when compared to that of at *z*_*L*_, whereas this behavior is reversed in case of diverging tapered artery. We can also observe that in case of nontapered artery the wall shear stress is same at both locations where the maximum height of the stenosis is occurring. This is because the annular radius varies with respect to the different locations of the maximum height of the stenosis.  Figure 9 shows the effect of velocity slip at both locations of the maximum height of the stenosis on wall shear stress. In general it is observed that shear stress at the wall is increasing as velocity slip at the wall is increasing. It is also observed that shear stress is more at the maximum height corresponding to the location *z*_*R*_. All further computations are done at the maximum height corresponding to *z*_*L*_.  Figure 10 shows the variation in shear stress at the wall for various values of the parameters *ϕ* and *m*. Here it is observed that the shear stress at the wall is increasing as the micropolar parameter is decreasing. It is also understood that for higher values of *m* the variation in the shear stress at the wall is less. The change in shear stress at the wall with respect to coupling number and tapered parameter is shown in Figure 11. From the figure it is clear that shear stress at the wall increases as the coupling number increases. The impact of catheter radius on wall shear stress at the maximum height of the stenosis is shown in Figure 12. Here catheter radius is significantly influencing shear stress at the wall and as catheter radius increases the physiological parameter also increases. In particular it is interesting to note that the wall shear stress is maximum in case of converging tapered artery and thick catheter.

**Figure 13:**
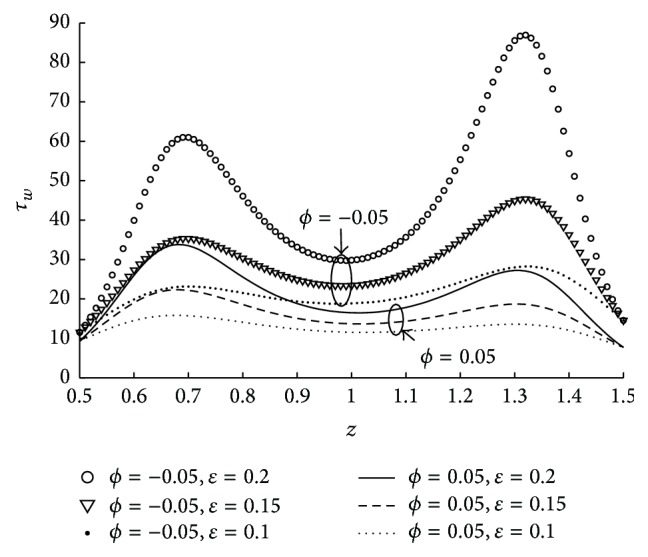
The variation of shear stress at wall in stenosed region with respect to *ϕ*, *ϵ* when *N* = 0.75, *r*_*c*_ = 0.1, *u* = 0.01, and *m* = 10.

**Figure 14:**
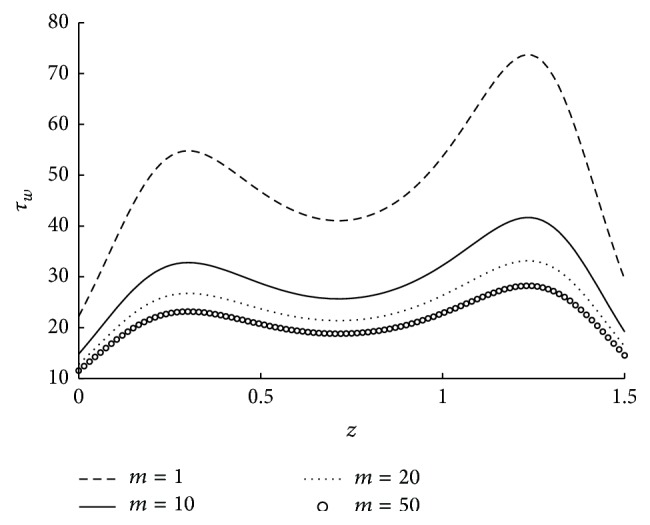
The variation of shear stress at wall in stenosed region with respect to *m* when *r*_*c*_ = 0.1, *u* = 0.01, *ϵ* = 0.1, *N* = 0.75, and *ϕ* = −0.05.

**Figure 15:**
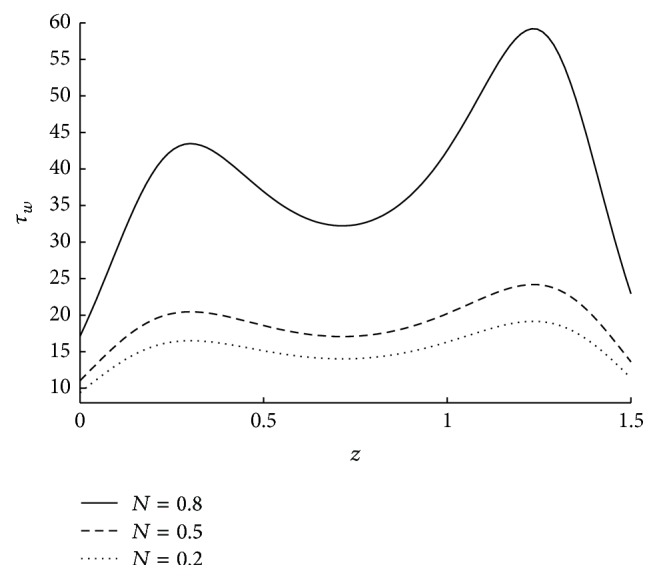
The variation of shear stress at wall in stenosed region with respect to *N* when *r*_*c*_ = 0.1, *ϵ* = 0.1, *u* = 0.01, *m* = 10, and *ϕ* = −0.05.

**Figure 16:**
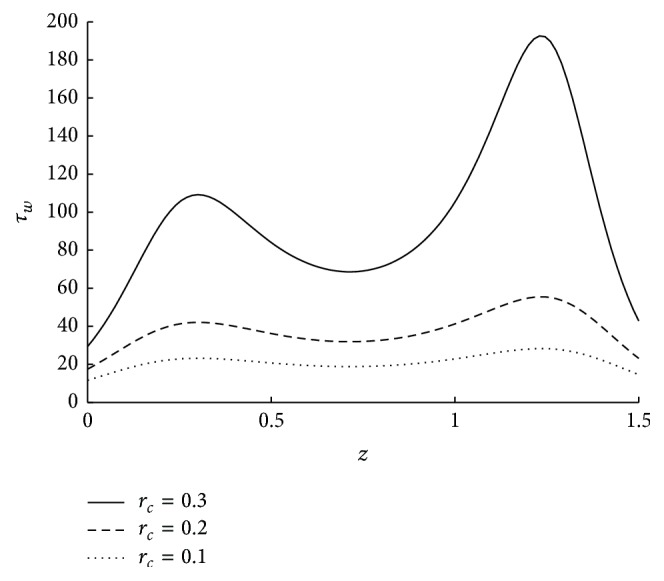
The variation of shear stress at wall in stenosed region with respect to *r*_*c*_ when *N* = 0.75, *ϵ* = 0.1, *u* = 0.01, *m* = 10, and *ϕ* = −0.05.

**Figure 17:**
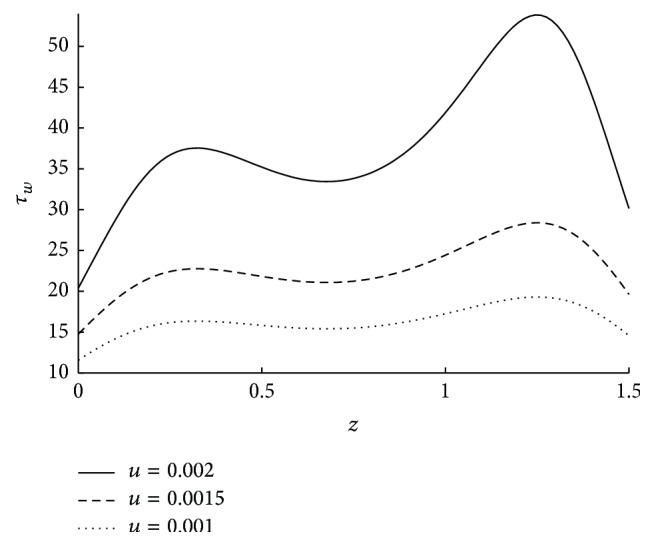
The variation of shear stress at wall in stenosed region with respect to *u* when *N* = 0.75, *r*_*c*_ = 0.1, *ϵ* = 0.1, *m* = 10, and *ϕ* = −0.05.

The variation of shear stress at the wall across the entire length of the stenosis for various values of the parameters have been discussed in the subsequent figures. The effect of tapered parameter (in both converging and diverging cases) and height of the stenosis on wall shear stress in the stenotic portion is depicted in Figure 13. The fact that the wall shear stress is more at *z*_*R*_ than *z*_*L*_ in converging case as discussed in Figure 8 is also confirmed here. Also the behavior is exactly reversed in case of diverging tapered artery. Further for a given *ϵ* the shear stress at the wall is more in converging artery than in diverging case. This is justified because in the converging case the fluid flow velocity is high in stenotic region than in the diverging case. Thus as *ϕ* increases shear stress at the wall decreases across the length of the stenosis. Also it is observed that as *ε* increases the shear stress at the wall across the entire length of the stenosis shows an increasing trend.  Figure 14 shows the wall shear stress distribution in the stenotic region for different values of the micropolar parameter and for the fixed values of the other parameters. As in the case of impedance, as the micropolar parameter is increasing the wall shear stress is decreasing. It can be further observed that initially when value of micropolar parameter is less, small change in *m* leads to significant change in *τ*_*w*_. However the variation in *τ*_*w*_ is very less even for a significant change in the values of *m* when *m* is high. This is because of very less coupling effects for higher values of *m*. The effect of coupling number on wall shear stress in the stenosed region is as in Figure 15. Here it is observed that, *τ*_*w*_ is increasing as *N* is increasing. *N* → 0 as the direct coupling of the microstructure to the velocity field, that is, *κ* goes to zero. Thus the decrease in coupling number decreases the wall shear stress. The influence of the radius of the catheter on wall shear stress is observed from the Figure 16. As the catheter radius increases the wall shear stress also increases. The effect of velocity slip on shear stress at the wall is sketched in Figure 17. As the velocity slip at the wall increases the stickiness at the wall reduces and velocity of the flow increases. This results in the high wall shear stress.

## 5. Conclusion

It is very important to understand the dynamics of blood flow in human physiological system. In view of this, a mathematical model is developed for axisymmetric flow of blood through overlapping stenotic tapered artery with velocity slip at the wall. Under the assumption of mild stenosis, closed form expressions for the axial velocity, microrotation vector, impedance and shear stress at the wall are obtained under appropriate boundary conditions. Via the described procedure flow conditions accounting for the presence of various parameters arising out of the fluid and geometry are discussed mathematically. Impedance and wall shear stress are numerically evaluated using Mathematica and Matlab.

(i) The locations of the extremum heights are different for different tapering angles. It is observed that impedance and wall shear stress differ significantly at these locations. In particular, impedance is maximum for converging tapered artery at *z*_*R*_. This was ignored by all the earlier authors to the best of our knowledge.
(ii) Impedance is increasing as the parameters *ϵ*, Γ, *N*, and *r*_*c*_ are increasing and the trend is reversed with respect to micropolar parameter and slip velocity.
(iii) The variation of shear stress at the wall is directly proportional to the coupling number, radius of the catheter, and slip velocity while it is inversely proportional with respect to *ϕ* and *m*.
(iv) It is that shear stress is increasing linearly for lower values of the *r*_*c*_, *N*, and *u* while it increases exponentialy for higher values of these parameters.
(v) The variation of shear stress across the entire length of the stenosis with respect to various parameters has also been studied. The results obtained are in predicted lines.
(vi) The results obtained have been validated with that of Back [4] and the pattern obtained is similar.
(vii) The impedance in case of overlapping stenosis is very high when compared to that of symmetric stenosis.

The mathematical treatment of the above phenomena is very realistic and is expected to be very useful in predicting the behavior of physiological parameters in the diagnosis of various arterial diseases and in the development of artificial organs.

## Figures and Tables

**Table 1:** 

*ϕ*	Location of *z*	*h*(*z*) for fixed value of *ϵ* = 0.1
−0.05	*z*_*L*_ = 0.7009	0.8392
	*z*_*C*_ = 0.9760	0.8756
	*z*_*R*_ = 1.3231	0.8080

0	*z*_*L*_ = 0.6882	0.8740
	*z*_*C*_ = 1.0000	0.9250
	*z*_*R*_ = 1.3118	0.8740

0.05	*z*_*L*_ = 0.6769	0.9081
	*z*_*C*_ = 1.0024	0.9752
	*z*_*R*_ = 1.2998	0.9393

**Table 2:** Validation with experimental and theoretical results.

Ratio of the diameter of the catheter to that of the vessel (in case of Back [4])/ratio of the radius of the catheter to that of the annular region (in the present study and as done by [14])	Impedance as obtained by Back [4] with 33% sugar-water solution with kinematic viscosity *ν* = 0.035 cm^2^ s^−1^	Impedance in case of symmetric stenosis as in [14] with *N* = 0.75, *m* = 50, Γ = 0.5 at *ϵ* = 0.1	Impedance in the present work with *N* = 0.75, *m* = 50, Γ = 0.5, *ζ* = 0 at *ϵ* = 0.1
0.001	1.17	37.1729	42.4983
0.01	1.28	41.0176	47.0415
0.1	1.74	60.4443	70.6899
0.2	2.35	92.4108	111.902
0.3	3.29	160.369	209.679
0.4	4.89	391.445	743.398
